# Building High Power Density of Sodium-Ion Batteries: Importance of Multidimensional Diffusion Pathways in Cathode Materials

**DOI:** 10.3389/fchem.2020.00152

**Published:** 2020-02-28

**Authors:** Mingzhe Chen, Yanyan Zhang, Guichuan Xing, Yuxin Tang

**Affiliations:** Institute of Applied Physics and Materials Engineering, University of Macau, Macau, China

**Keywords:** high power density, multidimensional diffusion pathways, cathode materials, sodium-ion batteries devices, materials design

## Abstract

Emerging sodium-ion batteries (SIBs) devices hold the promise to leapfrog over existing lithium-ion batteries technologies with respect to desirable power/energy densities and the abundant sodium sources on the earth. To this end, the discoveries on novel cathode materials with outstanding rate capabilities are being given high priority in the quest to achieve high power density SIBs devices, and the multi-dimensional Na^+^ migration pathways with low diffusion energy barriers are crucial. In light of this, the recent development of Prussian blue analogs and sodium superionic conductor (NASICON)-type materials with 3D Na^+^ diffusion pathways for building high power density NIBs are provided in this perspective. Ultimately, the future research directions to realize them for real applications are also discussed.

## Introduction

**Figure 1:**
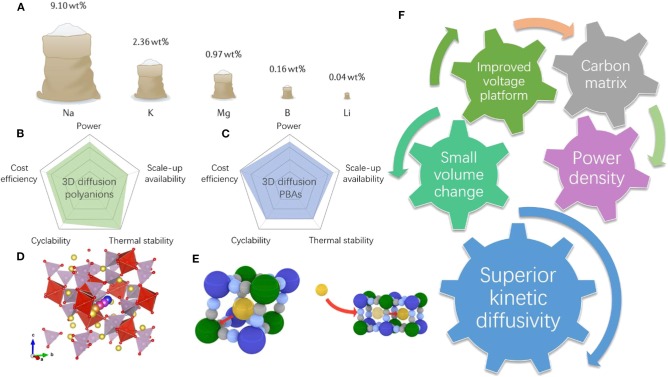
**(A)** Schematic illustrations of production and mining resources of elements that are used for batteries. Reproduced from paper (Vaalma et al., 2018) with permission from Springer Nature. **(B,C)** Evaluation and comparison of selected aspects of both 3D diffusion polyanions and PBAs, respectively. **(D)** Schematic illustrations of 3D diffusion pathways of polyanions and **(E)** PBAs. **(F)** The relevant properties and strategies of these two types of materials.

Although Li-ion batteries have received tremendous success in electronic and portable devices, the high price of lithium due to its limited and unequally distributed resources (Figure 1A) may hinder its further application in novel large-scale electrochemical energy storages systems (EESs) (Choi and Aurbach, 2016; Hwang et al., 2017; Vaalma et al., 2018). The rapid development of renewable energy sources such as wind energy, solar energy, and tide energy requires more cost-efficient and highly reliable large-scale EESs. SIBs have been widely considered as promising candidates for next-generation low-cost energy storage devices due to the almost unlimited resources of sodium (Li et al., 2017; Chen et al., 2019b,c). Due to the large atomic radius of Na (0.97 Å for Na^+^ and only 0.68 Å for Li^+^), however, hurdles that hinder the further applications of SIBs are mainly found in their sluggish sodium diffusion kinetics and large volume expansion, which are both critical parameters for revolutionary power density performance that can meet the real needs of power grids and large-scale EESs (Dai et al., 2017; Chen et al., 2019a). Therefore, it is important to explore novel cathode materials for SIBs with improved Na kinetics. From the solid-state diffusion perspective, the dimensions of diffusion pathways are the extremely important factors determining the diffusion coefficient of Na^+^ as well as the diffusion energy barriers. For facile and rapid ionic transportation, the cathode materials that possess three-dimensional (3D) Na^+^ migration pathways accompanied by low diffusion energy barriers are promising for building the high-power density SIBs (You et al., 2016; Chen S. et al., 2017). To this end, two emerging groups of Prussian blue analogs (PBAs) and sodium superionic conductor (NASICON)-type materials are recently developed. They both possess open 3D stable frameworks, exceptionally high ionic conductivities, and 3D sodium diffusion pathways. More importantly, these two types of materials usually undergo minimal volume changes during cycling, and their structures are highly tunable with different electrochemical properties (Lee et al., 2014; Wang B. et al., 2018). We compared the five important aspects for the real applications of these two types of materials in Figures 1B–E. We have witnessed an outstanding increase in publications regarding cathode materials with 3D diffusion pathways (Kim et al., 2012; You et al., 2016; Fedotov et al., 2018; Hurlbutt et al., 2018). Specifically, strategies such us nanosizing particles, introducing a uniform carbon coating/carbon matrix, and heteroatom doping are heavily used for better electrochemical properties (Figure 1F). These strategies have been developed to regulate the microstructures and surface atomic configurations to accelerate the charge transfer and kinetic properties as the same time.

In this perspective, we highlight (i) the specific key issues that are hindering the wider applications of these 3D Na^+^ diffusion pathway materials, (ii) the recent novel and potential strategies to achieve high-performance SIBs, and (iii) the approaches to achieve higher energy/power densities and long-term cycling stability, and (iv) the new emerging SIB devices. Our perspective sharps the future development of cathode materials for the high-power density of SIBs and provides effective methodologies for other related next-generation energy storage devices.

## PBAs With 3D Na^+^ Diffusion Pathways

Low-cost Na-hosting Prussian blue analogs usually possess the general structural formula of Na_*x*_M_2_[M_1_(CN)_6_]_*y*_·*n*H_2_O (0 ≤ *x* ≤ 2, 0 ≤ *y* ≤ 1), where M_1_ and M_2_ are transition metal such us Mn, Fe, Co, etc. (Li et al., 2015). Within this structure, PBAs allow various modifications of their chemical compositions without changing the overall crystalline structure. Normally, most of the studied PBAs occurs as cubic phases with 6-fold coordination of Fe^II, III^-O_6_ octahedra participating in the redox reactions (Xiao et al., 2015). Theoretically, PBAs allow a maximum number of two Na^+^ ions per formula unit if both the M_1_ and the M_2_ transition metal elements are electrochemically active, which can lead to a potential capacity over 170 mAh g^−1^, completely comparable with all other kinds of cathode material for SIBs (Wu et al., 2017). Such high capacity is not easy to achieve, however, since the defects, vacancies, and water molecules will greatly influence the crystal structures of PBAs. Their zeolitic water molecules tend to occupy the interstitial sites that are essential for the sodium ion diffusion. The ideal situation is that all the zeolitic water can be completely removed, while the coordination water can be retained to render structural stability. In addition, the poor intrinsic electronic conductivity of PBAs also remains a great challenge for better C-rate performance, although they possess 3D diffusion pathways. From the previous density function theory (DFT) calculations, the intercalation of Na^+^ with a larger ionic radius will incur the cost of increased insertion potential, since the steric interactions have a close correlation with the hydration energy. Both sodium and potassium possess sufficiently high redox potentials that an appropriate energy density can be secured. The role of water in ionic conductivity is still an open question for all researchers. A paddlewheel mechanism was previously proposed, in which the alkali metal ions move through channels and vacancies (Wessells et al., 2011). Coordinated water is beneficial to facilitate ion migration, while zeolitic water has a negative influence, obstructing improvement of ionic conductivity. Both computational and experimental insights into the effects of the presence of water in PBAs are urgently needed.

**Figure 2:**
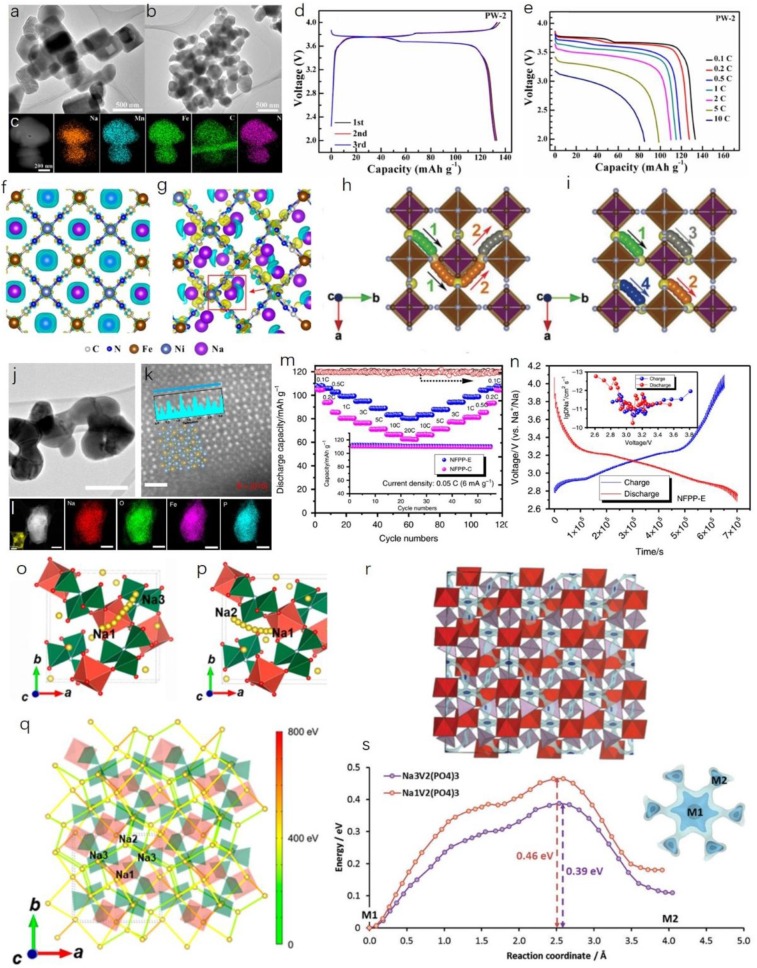
**(A,B)** Transmission electron microscope (TEM) images of Na-rich monoclinic phase Na_x_MnFe(CN)_6_. **(C)** Scanning TEM-energy dispersive spectroscopy (STEM-EDS) element mapping results for Na_*x*_MnFe(CN)_6_. **(D)** Charge-discharge profile and **(E)** C-rate performance of the as-obtained sample. Reproduced from paper (Shen et al., 2018) with permission from the American Chemical Society. Charge density analyses of **(F)** cubic-structured NiHCF and **(G)** rhombohedral-structured NiHCF. Reproduced from paper (Ji et al., 2016) with permission from the American Chemical Society. **(H,I)** Front view of possible 3D Na^+^ pathways between adjacent equivalent 24*d* sites. Reproduced from paper (You et al., 2016) with permission from WILEY-VCH. **(J)** Bright field (BF) image, **(K)** High-angle annular dark-field (HAADF) image with line profile in the inset, and **(L)** STEM-EDS mapping of as-obtained Na_4_Fe_3_(PO_4_)_2_(P_2_O_7_) (NFPP) nanoplate. **(M)** C-rate performance and cycling stability (inset) of NFPP-E nanoplate. **(N)** Galvanostatic intermittent titration technique (GITT) curve of NFPP-E electrode and corresponding calculated diffusion coefficient of Na^+^ ions. Reproduced from paper (Chen et al., 2019a) with permission from the Nature Publishing group. **(O,P)** Two typical 3D sodium diffusion pathways in Na_3_V(PO_3_)_3_N material and the **(Q)** corresponding activation barriers. Reproduced from paper (Kim et al., 2017) with permission from the American Chemical Society. **(R)** NASICON-type Na_3_V_2_(PO_4_)_3_ material with energy isosurface, showing interweaving of 3D Na^+^ pathways and **(S)** migration pathways of Na^+^ in the open 3D framework involving M1-M2-M1 hopping. Reproduced from paper (Wong et al., 2017) with permission from the Royal Society of Chemistry.

One promising approach is to synthesize and optimize the Na-rich phase, Prussian white. When M_2_ is Mn and M_1_ is Fe in Na_*x*_M_2_[M_1_(CN)_6_]_*y*_·*n*H_2_O (0 ≤ *x* ≤ 2, 0 ≤ *y* ≤ 1), a higher content of Na can be achieved. The valence of Fe is +2, however, and it is vulnerable to being oxidized to +3, so an inert atmosphere is required during both fabrication and centrifugal separation processes. By facile sodium citrate added precipitation method, Na-rich monoclinic phase Na_*x*_MnFe(CN)_6_ was successfully synthesized by Shen et al. (2018). Uniform microsize cuboid particles were obtained, and all the involved elements were uniformly distributed (Figures 2A–C). The high reversible capacity of 133.1 mAh g^−1^ was obtained, and satisfactory rate performance was achieved from 0.1 C to 10 C (1 C = 150 mA g^−1^), as shown in Figures 2D,E. The uniform cuboid particle morphology provides extra convenience for fast ion diffusion apart from the original 3D pathways. In recent years, rhombohedral PBAs have been receiving more and more attention, since the concentration of coordinated water can be reduced due to the rhombohedral lattice symmetry, which is particularly favorable for the Na-rich phases. A zero-strain nickel hexacyanoferrate (NiHCF) was introduced by Ji et al. (2016) using a simple coprecipitation method. It delivered a high operation voltage, good cycling performance, and superior C-rate capability. They found that the unexpected high operation voltage was mainly attributable to the asymmetric residence of Na^+^ ions along with the low charge density around Fe^2+^. Different charge density analyses indicated that the Na^+^ ions in cubic-structured NiHCF (c-NiHCF) tend to stay exactly in the center of interstitial channels (Figure 2F), while for rhombohedral-structured NiHCF (r-NiHCF), the Na^+^ ions prefer to asymmetrically stay at N-coordinated corners (Figure 2G). Also the Na-N distance in r-NiHCF is shorter than that in c-NiHCF. Dramatic electron polarization round N atoms occurs, leading to a charge redistribution between adjacent Ni^2+^ and Fe^2+^ ions.

Therefore, the operation voltage of r-NiHCF is slightly higher than that of c-NiHCF. This phenomenon also facilitates fast sodium diffusion with excellent C-rate performances. In addition, the open-channel 3D diffusion pathways can guarantee satisfactory low-temperature performance. You et al. synthesized the Na_2_FeFe(CN)_6_ materials with carbon nanotubes (CNT) to achieve potential low-temperature properties (You et al., 2016). The well-decorated CNT network provides excellent electrical contact with the current collector. In addition, they performed a DFT study and found that the evenly distributed enriched 24*d* sites can provide uniform 3D Na^+^ diffusion and feasible diffusion pathways, as shown in Figures 2H,I. There, PBAs are ideal candidates for low-temperature SIBs (Song et al., 2015; Peng et al., 2018). Further exploration should be focused on the inherent relationship between the crystal structure and the vacancies and water, and how these factors influence the sodium diffusion under various circumstances also needs to be urgently understood in detail.

## Polyanionic Compounds With 3D Na^+^ Diffusion Pathways

The polyanionic-type cathode materials are one of the important branches among all the kinds of available electrodes for SIBs. Their merits are obvious and distinctive, such as outstanding cycling stability, high safety, and suitable operating voltages (Lu et al., 2017; Chen et al., 2020). Most of the polyanionic compounds consist of the corner- or edge-sharing MO_6_ (*M* = transition metal) octahedra with connected XO_4_ (*X* = P, S, Si, etc.) tetrahedra. This unique structure can provide sufficient stability and ionicity around the MO_6_ octahedra, and, in turn, the ionic conductivity can be sustained within its 3D framework (Guo et al., 2017). Their sturdy framework can also render only small or even tiny volume shrinkage during cycling, since the topotactic reaction mechanism is dominant. Most of the polyanionic compounds are thermally stable due to their non-flammable nature (Ni et al., 2017). Also, polyanionic compounds normally possess high initial cycle Coulombic efficiency (ICE), which is an important parameter when making full cells. Nevertheless, most of the polyanionic compounds suffer from inherent low electronic conductivity, and therefore a well-decorated carbon coating and network are essential for their C-rate performance. According to the previous report, the majority of polyanionic composites possess one- or two-dimensional (1D or 2D) sodium diffusion pathways (Kim et al., 2013, 2015; Chen M. et al., 2017; Panigrahi et al., 2017; Chen et al., 2018; Fang et al., 2018; Zhu et al., 2019). The low-dimensional sodium diffusion pathways cannot guarantee satisfactory C-rate capabilities, especially in low temperature environments. Therefore, it is necessary to highlight those polyanionic compounds that possess unique 3D sodium diffusion pathways that are conducive toward real applications of SIBs and relevant energy storage devices. One class of these polyanionic composites comprises the sodium superionic conductor (NASICON)-type materials. Due to their high ionic conductivities, the NASICON-type materials also have been investigated as solid-state electrolytes (Li et al., 2018; Lu et al., 2018). Numerous papers have reported various NASICON-type materials, including both cathodes and anodes for SIB devices (Bui et al., 2015; Wang et al., 2017; Gao et al., 2018; Hu et al., 2018; Wang E. et al., 2018). Recently, we have introduced a new Fe-based polyanionic compound, Na_4_Fe_3_(PO_4_)_2_(P_2_O_7_), which can be classified as a new NASICON-type material (Chen et al., 2019a). With the help of ethylenediaminetetraacetic acid (EDTA) as the complexing agent in a sol-gel approach, the Na_4_Fe_3_(PO_4_)_2_(P_2_O_7_) nanoplates (denoted as NPFF-E) can be well-obtained with a high degree of crystallinity, as shown in Figures 2J,K. All the detected elements are uniformly distributed in one NPFF-E particle (Figure 2L). Outstanding C-rate performance can be obtained, and satisfactory cycling stability can be achieved as well (Figure 2M). In addition, we also determined the sodium diffusion coefficient through a galvanostatic intermittent titration technique (GITT) study. From Figure 2N, the sodium diffusion coefficients range from 10^−13^ to 10^−10^ cm^2^ s^−1^, which is highly competitive with other recognized NASICON-type materials. These high diffusion coefficients are critical for low-temperature performance.

Another newly recognized family of NASICON-type cathode materials with 3D sodium diffusion pathways comprises the V-based N-substituted polyanionic materials. Kang et al. recently reported a new member of the 4 V-class of zero-strain cathode materials, Na_3_V(PO_3_)_3_N, with unique 3D diffusion pathways for Na^+^ ions (Kim et al., 2017). One PO_3_N tetrahedron is connected to three VO_6_ octahedra, leading to a strong inductive effect which, in turn, influences the operation voltage during charge/discharge. The relevant sodium ion diffusion pathways and diffusion energy barriers were calculated via a DFT study, and illustrations are presented in Figures 2O–Q. This exciting discovery has opened up new interest in the high-voltage N-substituted cathode materials with unique unexpected 3D sodium diffusion pathways. As for the well-recognized NASICON-type Na_3_V_2_(PO_4_)_3_ material, great attention has been extensively paid to it due to its outstanding merits (high operation voltage, acceptable specific capacity, and excellent cyclability). Its 3D sodium diffusion properties have been well-investigated, with the 3D network involving M1-M2-M1 hops and low energy barriers. Each Na in an M1 site is actively connected to six M2 sites. The corresponding illustrations are shown in Figures 2R,S. In addition, we would like to see more polyanionic compounds discovered with 3D Na^+^ diffusion pathways in the search for advanced SIB storage devices with high power densities. It is suggested that further exploration should be concentrated on improving the reaction kinetics based on a more comprehensive understanding. Nevertheless, due to the intrinsic low electron conductivity of polyanionic material, how to maintain the well-designed carbon matrix or network and large-scale synthesis is still very challenging.

## Discussion and Perspectives

The ultimate goal for advanced sodium ion battery devices has never changed, that they should be thoroughly competitive with the lithium ion battery in energy density, power density, and lower overall manufacturing cost (Tang et al., 2017, 2018; Zhang et al., 2017, 2019; Guo et al., 2020). For full-cell sodium ion battery devices, the emphasis always falls on the cathode parts, since they possess key parameters such as high enough operation voltages, large theoretical capacity, and most of all, acceptable high-rate capability. Materials with 3D sodium diffusion pathways are a promising branch among all the reported ones, and their distinctive physical and chemical properties deserve more integrated investigations and a deeper and more comprehensive understanding. Both the PBAs and the polyanionic compounds with 3D sodium diffusion pathways have shown outstanding fast kinetics, leading to superior C-rate performance at various current densities. Further investigations are suggested to be focused on the improvements of their intrinsic kinetic properties as well as mass-production issues. Specifically, for PBAs, as mentioned above, the structure-kinetics-performance relationship is subject to the influence of each crystallographic component: binary carbon- and nitrogen-coordinated transition metal ions, the density distribution of electronic states at each sodium site, and the vacancies created by either zeolitic water or coordinated water. When dealing with the specific concerns about the actual Na^+^ diffusion channels and ionic conductivity, the bond length between carbon and carbon-coordinated transition metal elements is one of the key parameters for channel size, which, in turn, affects the ionic conductivity. The content of vacancies also has a strong influence on the Na^+^ diffusion channels and ionic conductivity. The appropriate amount of vacancies will provide extra channels for Na^+^ diffusion without cracking the local crystal structure. Nevertheless, how these parameters quantitatively influence the ionic conductivity as well as the crystal stability is still poorly understood. Another important issue that should not be neglected is the electronic conductivity. Up until now, only a few papers have focused on improving electronic conductivity directly by using the generalized gradient approximation with the Hubbard correction (GGA + U) method. Experimentally speaking, the electronic conductivity is strongly correlated with all the factors mentioned above and needs to be determined in an equational relationship. In the case of polyanionic compounds, the electronic conductivity is the most troublesome problem, and a well-designed carbon coating and carbon network are necessary even when the materials have high ionic conductivities. Therefore, how to maintain the delicate carbon network in a large-scale manufacturing procedure is a critical issue for the real application of polyanionic composites. Unlike the PBAs, only some of the reported materials possess 3D sodium diffusion pathways, although almost all the polyanionic compounds have open 3D frameworks. Compared to the PBAs, the activation energy barriers of these polyanionic compounds are slightly higher. Heteroatom doping can be considered as an effective approach to alter the pristine electron state density distributions around both MO_6_ octahedra and XO_4_ tetrahedra, which, in turn, may lower the charge barriers around each sodium site. The transition metal migration is also poorly understood to date [such as for Na_4_M_3_(PO_4_)_2_(P_2_O_7_) (M = Fe, Mn, etc.)], and how the phenomenon affects the related ion diffusion pathways and ionic conductivity should be determined in the future. It is also important to continuously discover more novel polyanionic cathode materials with 3D sodium diffusion pathways that have higher capacity and operation voltages. Besides, another important research direction is that how to combine both the PBAs and polyanions with the solid-state electrolyte properly so that their unique 3D diffusion properties can be more significantly magnified.

In Table S1, we summarize some of the reported cathodes for SIB devices with unique 3D diffusion pathways and their corresponding diffusion coefficients and activation barriers. These types of cathodes are especially important for high-power SIB devices for real applications. We can reasonably anticipate more technical breakthroughs, such as precise control of the water content in PBAs, ways to maintain a well-decorated carbon network at the industrial level, efficient organic/solid state electrolytes to fully achieve their theoretical capacities, and, in turn, the advanced SIB devices can be further designed and developed from a more technology based point of view.

## Data Availability Statement

The datasets generated for this study are available on request to the corresponding author.

## Author Contributions

MC, YZ, and GX prepared the manuscript. YT supervised the project.

### Conflict of Interest

The authors declare that the research was conducted in the absence of any commercial or financial relationships that could be construed as a potential conflict of interest.
