# Supplementary material for: Building High Power Density of Sodium-Ion Batteries: Importance of Multidimensional Diffusion Pathways in Cathode Materials
Source: Front Chem. 2020 Feb 28;8:152. doi: 10.3389/fchem.2020.00152 (PMC7058792; doi:10.3389/fchem.2020.00152)
Supplement: Supplementary file 1 [file Data_Sheet_1.PDF]

Supporting information

Building High Power Density of Sodium-Ion Batteries: Importance of Multidimensional Diffusion Pathways in Cathode Materials

Mingzhe Chen, Yanyan Zhang, Guichuan Xing, Yuxin Tang*

[1]Institute of Applied Physics and Materials Engineering, University of Macau, Macau, P. R. China

*** Correspondence:** yxtang@um.edu.mo
Keywords: high power density, multidimensional diffusion pathways, cathode materials, sodium-ion batteries devices, materials design

Table S1 Summary of kinetics parameters of reported 3D sodium diffusion cathodes for SIB devices.

Materials	Average Operation Voltage	Achievable Capacity (mAh g^{-1})	Rate Performance (mAh g^{-1})	Initial Coulombic Efficiency	Na^+ Diffusion - (cm^2 s^{-1})	Highest Activation barrier	Volume Expansion	Reference
**$Fe_4[Fe(CN)_6]_3$**	3.4 V	68 (10 mA g^{-1})	59 (480 mA g^{-1})	-	-	-	0.72 %	(Ji et al., 2016)
**$Na_2FeFe(CN)_6$**	2.9 V	170 (0.1 C)	120 (6 C)	90 %	10^{-10}–10^{-11}	0.64 eV	-	(You et al., 2016)
**$Na_2Mn[Mn(CN)_6]$**	2.6 V	209 (40 mA g^{-1})	157 (1 A g^{-1})	99.5 %	-	-	1.36 %	(Lee et al., 2014)
**$Na_2Mn[Fe(CN)_6]\cdot zH_2O$**	3.4 V	150 (14.3 mA g^{-1})	122 (2.86 A g^{-1})	87 %	-	-	-	(Song et al., 2015)
**$Na_{1.68}Ni_{0.3}Co_{0.7}[Fe(CN)_6]$**	3.3 V	143 (30 mA g^{-1})	88 (1.5 A g^{-1})	97 %	-	0.57 eV	3 %	(Peng et al., 2018)
**$Na_4Fe_3(PO_4)_2(P_2O_7)$**	3.2 V	113 (12 mA g^{-1})	80 (2.4 A g^{-1})	99 %	10^{-10}–10^{-13}	0.82 eV	4.0 %	(Chen et al., 2019)

**$Na_3V(PO_3)_3N$**	4.0 V	73 (14.6 mA g^{-1})	62 (0.73 A g^{-1})	-	-	0.8 eV	0.24 %	(Kim et al., 2017)
**$Na_4Mn_3(PO_4)_2(P_2O_7)$**	3.8 V	109 (0.05 C)	55 (20 C)	88 %	-	0.56 eV	7.0 %	(Kim et al., 2015)
**$Na_3V_2(PO_4)_3$**	-	-	-	-	10^{-10}–10^{-12}	0.6 eV	-	(Bui et al., 2015)
**$Na_3V_2(PO_4)_3$**	3.3 V	113 (0.5 C)	90 (10 C)	-	10^{-11}–10^{-12}	0.395 eV	7.6 %	(Li et al., 2018)
**$Na_3MnZr(PO_4)_3$**	3.8 V	107 (0.1 C)	50 (20 C)	87 %	-	-	5.5 %	(Gao et al., 2018)
**$Na_3MnTi(PO_4)_3$**	2.5 V	162 (0.2 C)	130 (2 C)	155 %	10^{-11}–10^{-13}	-	-	(Zhu et al., 2019)

References

Bui, K.M., Dinh, V.A., Okada, S., and Ohno, T. (2015). Hybrid functional study of the NASICON-type $Na_3V_2(PO_4)_3$: crystal and electronic structures, and polaron-Na vacancy complex diffusion. *Phys. Chem. Chem. Phys.* 17(45), 30433-30439. doi: 10.1039/c5cp05323d

Chen, M., Hua, W., Xiao, J., Cortie, D., Chen, W., Wang, E., et al. (2019). NASICON-type air-stable and all-climate cathode for sodium-ion batteries with low cost and high-power density. *Nat. Commun.* 10(1), 1480. doi: 10.1038/s41467-019-09170-5

Gao, H., Seymour, I. D., Xin, S., Xue, L., Henkelman, G., and Goodenough, J.B. (2018). $Na_3MnZr(PO_4)_3$: A High-Voltage Cathode for Sodium Batteries. *J. Am. Chem. Soc.* 140(51), 18192-18199. doi: 10.1021/jacs.8b11388

Ji, Z., Han, B., Liang, H., Zhou, C., Gao, Q., Xia, K., et al. (2016). On the Mechanism of the Improved Operation Voltage of Rhombohedral Nickel Hexacyanoferrate as Cathodes for Sodium-Ion Batteries. *ACS Appl. Mater. Interfaces* 8(49), 33619-33625. doi: 10.1021/acsami.6b11070

Kim, H., Yoon, G., Park, I., Park, K.-Y., Lee, B., Kim, J., et al. (2015). Anomalous Jahn–Teller behavior in a manganese-based mixed-phosphate cathode for sodium ion batteries. *Energy Environ. Sci.* 8(11), 3325-3335. doi: 10.1039/c5ee01876e

Kim, J., Yoon, G., Lee, M.H., Kim, H., Lee, S., and Kang, K. (2017). New 4V-Class and Zero-Strain Cathode Material for Na-Ion Batteries. *Chem. Mater.* 29(18), 7826-7832. doi: 10.1021/acs.chemmater.7b02477

Lee, H.-W., Wang, R.Y., Pasta, M., Lee, S.W., Liu, N., and Cui, Y. (2014). Manganese hexacyanomanganate open framework as a high-capacity positive electrode material for sodium-ion batteries. *Nat. Commun.* 5, 5280. doi: 10.1038/ncomms6280

Li, X., Huang, Y., Wang, J., Miao, L., Li, Y., Liu, Y., et al. (2018). High valence Mo-doped $Na_3V_2(PO_4)_3$/C as a high rate and stable cycle-life cathode for sodium battery. *J. Mater. Chem. A* 6(4), 1390-1396. doi: 10.1039/c7ta08970h

Peng, J., Wang, J., Yi, H., Hu, W., Yu, Y., Yin, J., et al. (2018). A Dual-Insertion Type Sodium-Ion Full Cell Based on High-Quality Ternary-Metal Prussian Blue Analogs. *Adv. Energy Mater.* 8(11), 1702856. doi: 10.1002/aenm.201702856

Song, J., Wang, L., Lu, Y., Liu, J., Guo, B., Xiao, P., et al. (2015). Removal of Interstitial H_2O in Hexacyanometallates for A Superior Cathode of A Sodium-ion Battery. *J Am. Chem. Soc.* 137(7), 2658-2664. doi: 10.1021/ja512383b

You, Y., Yao, H.R., Xin, S., Yin, Y.X., Zuo, T. T., Yang, C.P., et al. (2016). Subzero-Temperature Cathode for a Sodium-Ion Battery. *Adv. Mater.* 28(33), 7243-7248. doi: 10.1002/adma.201600846

Zhu, T., Hu, P., Wang, X., Liu, Z., Luo, W., Owusu, K.A., et al. (2019). Realizing Three-Electron Redox Reactions in NASICON-Structured $Na_3MnTi(PO_4)_3$ for Sodium-Ion Batteries. *Adv. Energy Mater.* 9(9), 1803436. doi: 10.1002/aenm.201803436
